# Evaluating Diabetes Mobile Applications for Health Literate Designs and Functionality, 2014

**DOI:** 10.5888/pcd12.140433

**Published:** 2015-05-07

**Authors:** Charlene A. Caburnay, Kaitlin Graff, Jenine K. Harris, Amy McQueen, Madeleine Smith, Maggie Fairchild, Matthew W. Kreuter

**Affiliations:** Affiliations: Kaitlin Graff, Amy McQueen, Madeleine Smith, Maggie Fairchild, Health Communication Research Laboratory, George Warren Brown School of Social Work, Washington University in St. Louis; Jenine K. Harris, George Warren Brown School of Social Work, and Washington University Center for Diabetes Translation Research, Washington University in St Louis; Matthew W. Kreuter, Health Communication Research Laboratory, George Warren Brown School of Social Work, and Washington University Center for Diabetes Translation Research, Washington University in St Louis, St Louis, Missouri.

## Abstract

**Introduction:**

The expansion of mobile health technologies, particularly for diabetes-related applications (apps), grew exponentially in the past decade. This study sought to examine the extent to which current mobile apps for diabetes have health literate features recommended by participants in an Institute of Medicine Roundtable and compare the health literate features by app cost (free or not).

**Methods:**

We used diabetes-related keywords to identify diabetes-related apps for iOS devices. A random sample of 110 apps (24% of total number of apps identified) was selected for coding. The coding scheme was adapted from the discussion paper produced by participants in the Institute of Medicine Roundtable.

**Results:**

Most diabetes apps in this sample addressed diabetes management and therapeutics, and paid apps were more likely than free apps to use plain language strategies, to label links clearly, and to have at least 1 feature (a “back” button) that helps with the organization.

**Conclusion:**

Paid apps were more likely than free apps to use strategies that should be more useful and engaging for people with low health literacy. Future work can investigate ways to make free diabetes mobile apps more user-friendly and accessible.

## Introduction

The expansion of health care technologies, particularly mobile health technologies, grew exponentially in the past 10 years (1). Mobile health (or mHealth) is a subset of eHealth, defined as “the use of mobile computing and communication technologies in health care and public health” (2). The expansion of mHealth applications (apps) is also documented. A systematic review of mHealth research showed a surge in the number of scientific articles on this topic from 2005 through 2011 (1). More than half of the 352 studies involved testing of a mobile app, and most (86%) studies applied a quantitative methodology (1).

The rising availability of these mobile technologies corresponds with an increase in ownership of mobile telephones and tablets and the use of apps. National surveys in 2013 found that 34% of US adults owned a tablet and 91% owned a cellular telephone (1,3). Of cellular telephone owners, 55% had a smartphone (4) and 50% downloaded apps on their phone, an increase from 22% in 2009 (5). Although smartphone and tablet ownership has increased in nearly every major demographic group, ownership varies by income and age group (3,4). For example, younger adults, regardless of income, are likely to own a smartphone, whereas older adults who own a smartphone are more likely to be in upper income levels (4).

The review of mHealth apps (1) found that, in general, mHealth research focused predominantly on chronic conditions, and of these conditions, diabetes was the most frequent focus (1). A recent review of diabetes-specific apps available in 2013 for the iOS and Android operating systems (6) evaluated range of functions, target audiences, languages, cost, ratings, interfaces, and usability and found that apps had moderate to good usability among older (aged 50 or older) adults who had diabetes when the apps had a small range of functions. The review emphasized that simplicity of use and understandable terminology is especially important for maximum usability among older populations.

The most common types of diabetes apps identified were for health tracking or self-monitoring tasks such as recording blood glucose levels, insulin levels, and medication use (7–9). Other types of apps were insulin-dose calculators (7,8), physician-directed apps, food reference databases, social forums or blogs, and exercise apps (7). These diabetes apps did not demonstrate clinical effectiveness or integrate with health care delivery systems; other limitations included potential threats to safety and privacy, usability issues, and lack of personal feedback (7,9,10).

Although the surge in the development of diabetes apps and smartphone ownership continues, it is questionable how relevant and appropriate these types of health information technologies are for people with low health literacy — people who have a limited ability to obtain, process, and understand basic health information for making health decisions (11). In general, those with low health literacy are less likely to understand health information and know how to access and obtain prevention services (11–13), have worse glycemic control (14), and have higher rates of diabetes complications (14).

People with low health literacy are less likely to access and use health information technology (15) or be computer literate (16). Having eHealth literacy promotes self-efficacy for, and use of, health apps (17). Reviews of mobile apps (18) recommend certain design features to increase effort, efficiency, and satisfaction among low-literacy users, including the following: text-based interfaces (19); graphical cues, including bigger widgets (18,19); language support in text and audio (18); “back” and “home” buttons (19); linear navigation (navigation bar and scrollbars) and minimal hierarchical structures (18,19); and avoidance of nonnumeric text input and scrolling menus (18).

A 2013 Institute of Medicine (IOM) Roundtable on Health Literacy’s Collaborative on New Technologies yielded a discussion paper in which the authors summarize and suggest strategies for improving health literacy and usability in the development of health literate apps (20). These strategies are based on those developed by the US Department of Health and Human Services (21) and adapted for the development of mHealth apps. These strategies, along with actions (20), include the following:

- learning about users — identifying users and what they are trying to do and engaging them in the design process;
- writing actionable content — putting the most important information first, staying positive and realistic, providing action steps, and writing in plain language;
- displaying content clearly — using short paragraphs, large font size, white space, and clear labels;
- organizing and simplifying — using labels and providing easy access to home pages, linear information paths, and search and browse functionality;
- engaging users — including printer-friendly tools, simplified controls and buttons, and interactive content; and
- evaluating and revising the site — using experienced moderators to test the site with users of low literacy and low health literacy.

These recommendations were developed for the creation of apps, but they have not yet been used to evaluate existing diabetes-related mHealth apps. The objective of this study was to evaluate diabetes-related mHealth apps according to the recommendations of the IOM Roundtable discussion paper (20). Additionally, because the cost of an app may lead a user to select a free app instead of an app that has even a minimal cost, we compared the health literate features of diabetes apps by app cost (free or not).

## Methods

### Sample

The keywords “diabetes,” “diabetic,” “type 1 diabetes,” and “type 2 diabetes” were entered into the search field of the Apple App Store in April 2014 to identify English-language diabetes-related apps for iOS devices (iPad and iPhone). Our search yielded 460 apps that contained keywords in the app name, description, or reviews. We randomly selected 110 (24%) apps (Appendix).

### Coding

Coders first downloaded each app to an iPad and familiarized themselves with the app’s features. Next, coders entered information for each app into an electronic database. Apps were coded for general characteristics listed in the App Store. Then, coders recorded diabetes-related content and recommendations for designing health literate mobile apps as published in the IOM discussion paper (20). Trained research assistants first conducted the coding in teams while training and then coded individually. Intercoder reliability was calculated on the basis of a 10% sample and indicated substantial agreement (22): κ = 0.77 (95% confidence interval, 0.71–0.83). Each of 4 coders analyzed approximately 30 apps.

#### General characteristics

The Apple App Store provided the following data for each app: title, price, age rating, category, the total number of ratings provided by app users, and the number of stars (star rating), which ranged from 1 star to 5 stars (with 5 being the highest rating). The age rating was classified as 4 years or older (no objectionable material); 12 years or older (mild language, frequent/intense/realistic violence, and mild or infrequent mature or suggestive content not suitable for those <12 y); or 17 years or older (must be 17 years old to purchase, may contain frequent and intense offensive language, violence, or mature themes not suitable for those <17 y). The app category included health and fitness, medical, food and drink, education, lifestyle, social networking, business, reference, and utilities.

#### Public health variables

The research team developed categories for 3 public health variables and 4 health literate design strategies. For each variable, coders could select multiple categories (ie, categories were not mutually exclusive). The public health variables coded were type of diabetes, diabetes continuum, and app focus. The type of diabetes was mentioned in the app description or on the app itself and was categorized as type 1 diabetes, type 2 diabetes, prediabetes, gestational diabetes, “not specified,” or “other.” “Diabetes continuum” refers to the stage of diabetes-related behavior targeted by the app. We developed the following categories for state of behavior: prevention (eg, healthy eating, exercising); screening, diagnosis, or symptoms of diabetes (eg, getting a blood glucose check for a diagnosis of diabetes or symptoms of diabetes); management or therapeutics (eg, checking blood glucose regularly, eating healthy and exercising, preventing complications); or none (ie, no diabetes behaviors mentioned). “App focus” refers to the diabetes-related focus of the app, including primary prevention (eg, health promotion activities such as eating fruits and vegetables and regular exercise); screening (eg, blood glucose check to screen for diabetes); symptoms or diagnosis (eg, confirming diagnosis of diabetes through a blood glucose test, identifying diabetes by signs or symptoms); management or therapeutics (eg, insulin therapy for the regulation of blood glucose, lifestyle changes); complications (eg, the biological consequences of untreated diabetes, risk factors for complications); and research, science, and technology (eg, technology developed to manage or prevent diabetes).

The health literate design strategies (20) assessed by coders were 1) writing in plain language (eg, using common, everyday words; using personal pronouns such as “you”; avoiding undefined technical or medical terms; using active voice, action words, and present tense; keeping sentences short); 2) displaying content clearly (eg, limiting paragraph size by using bullets and short lists, labeling links for images and descriptions, using images that facilitate learning, using bold colors with contrast, avoiding dark backgrounds); 3) organizing and simplifying the app (eg, easy access to a homepage or menu page; a “back” button; the ability to search and browse; integration with email, calendar, and maps); and 4) engaging users* (*eg, printer-friendly tools and resources; interactive content; audio and visual features; connections to new media such as Twitter or text messaging).

We used SPSS v.20 (IBM Corporation) to calculate descriptive statistics and *t* tests to identify associations between app characteristics and price (free vs not free). Significance was determined at a level of α = .05 for 2-tailed tests.

## Results

Of the 110 apps, 76 (69%) were free (Table). General characteristics were not significantly different according to whether the app was free or not. The 34 apps that were not free ranged in price from $0.99 to $29.99, with a mean price of $4.57 (interquartile range, $1.99–$5.99). User ratings were provided for 65 apps; the average number of ratings per app was 148, and the average star rating was 3.4. Most apps (78.2%) were rated for ages 4 years or older, and the categories in which the apps were placed most often were health and fitness (43.6%) and medical (43.6%).

**Table:** Characteristics of Free and Paid Diabetes-Related Apps, by Cost (Free or Paid For)^a^

Characteristic	All Apps (n = 110)	Free Apps (n = 76)	Paid Apps (n = 34)	*P* Value^b^
**General^c^**				
**No. of ratings for each app, mean (SD)**	148.3 (334.7)	125.9 (337.6)	211.8 (328.0)	.37
**Star rating,^d^ mean (SD), n**	3.4 (1.0)	3.4 (1.1)	3.6 (0.8)	.45
**Age rating^e^**				
≥4 y	86 (78.2)	60 (78.9)	26 (76.5)	.74
≥12 y	9 (8.2)	5 (6.6)	4 (11.8)	
≥17 y	1 (0.9)	1 (1.3)	0	
Not listed	12 (12.7)	10 (13.2)	4 (11.8)	
**App category**				
Health and fitness	48 (43.6)	33 (43.4)	15 (44.1)	.68
Medical	48 (43.6)	32 (42.1)	16 (47.1)	
Food and drink	5 (4.5)	4 (5.3)	1 (2.9)	
Education	2 (1.8)	1 (1.3)	1 (2.9)	
Lifestyle	2 (1.8)	2 (2.6)	0	
Social networking	2 (1.8)	2 (2.6)	0	
Business	1 (0.9)	1 (1.3)	0	
Reference	1 (0.9)	0	1 (2.9)	
Utilities	1 (0.9)	1 (1.3)	0	
**Public health variable**				
**Type of diabetes mentioned in app**				
Type 1	5 (4.5)	5 (6.6)	0	.13
Type 2	5 (4.5)	4 (5.3)	1 (2.9)	.59
Prediabetes	1 (0.9)	1 (1.3)	0	.50
Gestational diabetes	2 (1.8)	1 (1.3)	1 (2.9)	.56
Not specified	96 (87.3)	64 (84.2)	32 (94.1)	.15
Other	3 (2.7)	3 (3.9)	0	.24
**Diabetes continuum**				
Prevention	38 (33.3)	26 (33.8)	12 (32.4)	.89
Screening, diagnosis, symptoms	12 (10.5)	9 (11.7)	3 (8.1)	.56
Management, therapeutics	84 (73.7)	56 (72.7)	28 (75.7)	.74
None	14 (12.3)	12 (15.6)	2 (5.4)	.12
**App focus**				
Primary prevention	34 (30.9)	23 (30.3)	11 (32.4)	.83
Screening	9 (8.2)	6 (7.9)	3 (8.8)	.87
Diagnosis or symptoms	9 (8.2)	5 (6.6)	4 (11.8)	.36
Management or therapeutics	73 (66.4)	51 (67.1)	22 (64.7)	.81
Complications	14 (12.7)	10 (13.2)	4 (11.8)	.84
Research, science, technology	10 (9.1)	7 (9.2)	3 (8.8)	.95
Other focus	9 (8.2)	9 (11.8)	0	.04
**Health literate design strategies**				
**Use plain language**				
Use common, everyday words	88 (80.0)	57 (75.0)	31 (91.2)	.05
Use personal pronouns	54 (49.5)	34 (45.3)	20 (58.8)	.19
Avoid undefined technical or medical terms	79 (71.8)	50 (65.8)	29 (85.3)	.04
Use active voice	82 (74.5)	52 (68.4)	30 (88.2)	.03
Use action words	83 (75.5)	53 (69.7)	30 (88.2)	.04
Use present tense	89 (80.9)	57 (75.0)	32 (94.1)	.02
Keep sentences short	93 (84.5)	61 (80.3)	32 (94.1)	.06
**Display content clearly**				
Links labeled clearly	102 (92.7)	68 (89.5)	34 (100.0)	.05
Images facilitate learning	34 (30.9)	23 (30.3)	11 (32.4)	.83
Use bold colors with contrast	89 (80.9)	60 (78.9)	29 (85.3)	.43
**Organize and simplify**				
Easy access to a homepage	80 (73.4)	51 (68.0)	29 (85.3)	.06
Easy access to a menu page	63 (57.8)	39 (52.0)	24 (70.6)	.07
Has a “back” button	90 (81.8)	57 (75.0)	33 (97.1)	.01
Search and browse	53 (48.2)	34 (44.7)	19 (55.9)	.28
Integrates with email	39 (35.5)	23 (30.3)	16 (47.1)	.09
Integrates with calendar	9 (8.2)	4 (5.3)	5 (14.7)	.10
Integrates with maps/GPS	7 (6.4)	5 (6.6)	2 (5.9)	.89
**Engage users**				
Printer-friendly tools and resources	11 (10.0)	6 (7.9)	5 (14.7)	.27
Include interactive content that users can tailor	77 (70.0)	51 (67.1)	26 (76.5)	.32
Incorporate audio and visual features	17 (15.5)	13 (17.1)	4 (11.8)	.47
Explore new media such as Twitter or text messaging	18 (16.4)	14 (18.4)	4 (11.8)	.38

Abbreviation: SD, standard deviation.

a All values are number (percentage) unless otherwise indicated.

b Determined by *t* test (number of ratings and star rating) or χ^2^ test; α = .05 for 2-tailed tests.

c The number of ratings and star rating were based on the following n’s: for all apps, n = 65; for free apps, n = 48; for paid apps, n = 17.

d Range is 1 to 5 stars, with 5 being the highest rating.

e The age rating was classified as 4 years or older (no objectionable material); 12 years or older (mild language, frequent/intense/realistic violence, and mild or infrequent mature or suggestive content not suitable for those <12 y); or 17 years or older (must be 17 years old to purchase, may contain frequent and intense offensive language, violence, or mature themes not suitable for those <17 y).

None of the public health variables were significantly different according to whether the app was free or not. Most (87.3%) apps did not specify diabetes type. Only 5 apps specified type 1 diabetes, and only 5 apps specified type 2 diabetes. Across the diabetes continuum, most (73.7%) apps addressed behaviors related to diabetes management or therapeutics, and a third (33.3%) addressed prevention. Other apps addressed diabetes screening, diagnosis, or symptoms (10.5%), and 12.3% did not address any stage on the continuum. Consistent with our findings on continuum, 66.4% of apps focused on management or therapeutics, and 30.9% focused on primary prevention.

**Using plain language.** Across all plain language strategies combined, 84% of apps used at least 1 strategy, and this finding did not differ by whether the app was free or not. However, we found significant differences between the 2 types of apps according to several strategies. Paid diabetes apps were significantly more likely to use common, everyday words (91.2%, paid vs 75.0%, free; *P* = .05); avoid undefined technical or medical terms (85.3% vs 65.8%; *P* = .04); and use active voice (88.2% vs 68.4%; *P* = .03), action words (88.2% vs 69.7%; *P* = .04), and present tense (94.1% vs 75.0%; *P* = .02).

**Displaying content clearly.** Paid apps were more likely than free apps to label links clearly (100.0% vs 89.5%, *P* = .05). Of both kinds of apps, most (80.9%) used bold colors with contrast but only 30.9% used images that facilitated learning; we found no differences between free and paid apps for these strategies.

**Organizing and simplifying.** Most (83.6%) apps provided easy access to a homepage or menu page; 85.3% of paid apps had a homepage, whereas 68.0% of free apps had one (*P* = .06); 70.6% of paid apps had easy access to a menu page, whereas 52.0% of free apps had one (*P* = .07). Paid apps were significantly more likely to include a “back” button (97.1% vs 75.0%, *P* = .006). No other differences between free and paid apps were found for other organizing strategies. Less than half (44%) of all apps were integrated with other applications (email, calendar, or maps).

**Engaging users.** Most (70.0%) apps had interactive content that users could tailor, but otherwise, less than 17% of apps had other engaging features such as printer-friendly tools, audio and visual features, or integration with new media such as Twitter or texting.

## Discussion

In this study, a sample of diabetes-related apps was coded for public health characteristics and health literate design strategies for mHealth apps. These apps were rated highly by users, and most were classified as appropriate for children and adults. Consistent with other studies of health apps and diabetes apps, most of the diabetes apps in this sample addressed diabetes management and therapeutics (7–9). Paid apps were more likely than free apps to use health literate design strategies such as using plain language, labeling links clearly, and having a “back” button to help with organization.

One explanation for these differences is that with paid apps, perhaps more effort was undertaken to conduct formative research and usability testing before product launch. Those activities may have identified functions in the app for which the user experience could be improved to increase understanding and ease of use.

Because low health literacy is more likely among people of low socioeconomic status (23), the cost of apps may be prohibitive for people with low health literacy. If these people are more likely to use free diabetes apps, then they are more likely to have apps that lack features that enhance usability and understanding. Further research can identify diabetes app characteristics, including functionality, cost, and ratings, that may influence potential users to pay for an app instead of downloading one for free. Because user ratings of free apps and paid apps did not differ significantly, it would also be helpful to conduct usability tests to directly compare levels of satisfaction for free and paid apps. Also, by understanding which types of diabetes apps people with low literacy would choose to use regardless of cost, we could identify where and how resources for improving the health literacy of mobile diabetes apps would be best used.

Our study has several limitations. Although we used *Health Literacy Online* (21) as a tool to rate existing apps, its original purpose was to help guide the design of health websites, including strategies for testing usability. Not all *Health Literacy Online* strategies and actions, such as those requiring knowledge of the app developers’ target users and usability processes, were included in the codebook for our study because of a lack of information. Because the information coded in our study could be ascertained only by viewing the app description and ratings information and using the downloaded app, we did not have enough background information to know the history of each app’s development or what, if any, usability testing was done before its launch. *Health Literacy Online* is one of several tools that can help guide the creation of health literate mHealth applications or to assess the health literacy of health information materials, including those that are digitally based (eg, mobile apps, websites, computer applications) (24,25). Using this tool for existing apps may not be as appropriate or useful for usability outcomes, but it is a starting point to help evaluate the health literacy of diabetes apps.

Another limitation of this study is the generalizability of the sample of diabetes apps selected. The use of a simple random sampling strategy yielded 4 of the top 10 most popular diabetes apps in the App Store (as of February 2015). In addition, the search terms included only diabetes-related terms, because we were interested only in apps that self-identified as diabetes-related through the app name or description. Other search terms such as “glucose” or “blood sugar” were not used. However, an additional search using the terms “glucose” or “blood sugar” yielded 294 apps, 176 of which also appeared on the list of diabetes-related apps.

Finally, the sample of apps examined included only iOS apps and did not include any Android-compatible apps. Because African American cellular telephone owners are more likely than whites or Latinos to own an Android telephone instead of an iPhone (42%, African Americans; 26%, whites; 27%, Latinos) (4), this may limit the study’s applicability to more diverse audiences.

In general, the findings of this study indicate that additional work should be done to improve mHealth apps. In particular, encouraging a development process for free diabetes-related apps to make them more user-friendly and accessible to diverse audiences could potentially increase their use and understandability among audiences, especially people with low health literacy.

## Appendix. Apps in Final Sample

1000 Diabetes Dictionary

150 Healthy Foods

A1cConverter

AADE13 mobile

AAP Essentials: Type 2 Diabetes

bant — A diabetes app for the ePatient

Best Diabetes Control Lite

BigAppleRx

Blausen Human Atlas Lite

Blood Sugar Diabetes Control

BloodWork Lite

BMI Calculator Free

California Health Care Report Card

Calorie Counter Pro by MyNetDiary

CalorieKing Food Search

Carb Manager — low carbohydrate diet tracker

Carb Master — Daily Carbohydrate Tracker

Carburetor-Diabetes

Care After Kidney Transplant

CDC eCards

ControlMyWeight — Calorie Counter

Daily Carb Premium

Dexcom Guide

Diabetes Guide & Lowering Tips

Diabetes Health Mobile

Diabetes in Pregnancy — Gestational Diabetes Logbook, Diabetes Manager, Pregnancy Diabetes Tracker

Diabetes Information

Diabetes Log

Diabetes Pacer with Glucose Diary, Pedometer, Auto Step Tracking, Weight Tracker

Diabetes Risk

Diabetes Risk Calculator

Diabetes Risk Score

Diabetes Support Forum

Diabetes Terms

Diabetes Tracker with Blood Glucose/Carb Log by MyNet Diary

Diabetes Trivia Quiz — The Fun Medical Game For Healthy Diabetics

Diabetic Connect

Diabetic Friendly Recipes

Diabetic patients, follow and monitor your glucose levels in the blood (SMBG)

Diabettes

Diamedic

Digital Health Scorecard

DocGuide

Drag n’ Cook

Dressing Selection

DrugInfoLine

Easy Diabetes Pro

Emergency Contact

Emergency First Aid — Instant Sel

Food Sense

FoodSmart — Diet, Calories and Healthy Grocery Shopping List

Glucagon

Gluco Logger by APG Solutions

Gluco Share

Glucose Companion

Glucose Tracker Lite — Log and Monitor Your Blood Glucose Levels

Healing chants and mantras

Health Care Quality Matters

Healthspek Viewer — PHR

Healthy Diet & Gluten Free, Allergy, GMO Scanner by NxtNutrio

Healthy Heart 2

Healthy Life Labs A1c Converter

HoMedics

iBolusCalc Diabetes Blood

iCalCalc LITE

iCook Recipes

iJoggingLite

Insulin Calculator

Insulin To Carb (I:C) Ratio

JDRF Walk to Cure Diabetes

JDRF-TELUS Walk to Cure

Johns Hopkins ABX Guide 2014

Johns Hopkins ABX, HIV, Diabetes Guides with Updates

Living well with Diabetes

Lumen Trails Food+ A Universal App That Lets You Create a Food Diary, Nutrition Plan, Fitness Tracker, Diabetes Journal

MaculaTester

Managing Type 1 Diabetes: A guide for kids and their families

Medical Benefits of Fruits

MedicalMe

MediCarer

MOWA-Mobile Wound Analyze

My Diet

My Diet Diary — Calorie Counter, Weight Log, Exercise and Fitness Tracker, Food and Nutrition Journal for Calorie Watchers

myMedtronic Connect

Natural Treatments

Nephrology News

Novo Nordisk HbA1c

NutriCHX

NutriGuides

OnTimeRx FREE

Pacer-Pedometer plus Weight Management and Blood Pressure Tracker

Paediatric Emergencies Lite

PillManager

Pocket A1c

Pocket Dietitian

Power 20 Fitness Trainer Free 20-Minute Daily Workout

Pumps4kids

RapidCalc Diabetes Manager

Recipes For Diabetes

Salad Recipes Free

SAT ATP III Lipid Management

Shot In The Arm

SiDiary

Simple Diabetes

Sleep Assess

StandApp Pro

TopTenDiabetes

Track Your Blood Sugar Level

URIGHT Diabetes Manager

Weight Track
